# Integrating Big Five Personality Traits and TPACK: A Pedagogical Framework for Collaborative Learning Management System (C-LMS) in Higher Education

**DOI:** 10.12688/f1000research.183549.1

**Published:** 2026-08-04

**Authors:** Daniel Lai, Sook Ling Lew, Shih Yin Ooi

**Affiliations:** 1 Multimedia University Faculty of Information Science and Technology, Ayer Keroh, Melaka, Malaysia; 2 Centre for Advanced Analytics, CoE for Artificial Intelligence, Faculty of Information Science and Technology, Multimedia University, Ayer Keroh, Melaka, Malaysia

**Keywords:** Big Five Personality Traits, TPACK, Collaborative Learning, Pedagogical Framework, Educational Technology, Learning Management System

## Abstract

**Background:**

The current research assesses whether students can achieve better academic performance through the proposed collaborative learning management system (C-LMS) developed as the primary virtual collaborative learning environment. It is intended to examine the relationship between students’ personality traits and their technological, pedagogical, and content knowledge (TPACK) proficiency and to determine their continued use of technology.

**Methods:**

The current research initiated an investigation of the issue from psychological perspectives, using students’ Big Five Personality Traits (BFPT) as predictors and extending the study by grounding it in the TPACK framework. It aimed to explain the relationship between BFPT and TPACK among students regarding their continued use of technology. The research experiment was conducted using the developed proposed C-LMS integrated into the virtual collaborative learning environment. Participants completed the provided pre-module knowledge-checking test after completing the BFPT questionnaire. Then, they needed to complete a post-module knowledge-checking test, a TPACK questionnaire, and course feedback. Their course feedback indicates their intention to continue using technology.

**Results:**

Based on the findings, the students’ BFPT had a significant effect on their TPACK and continued use of technology, except for extraversion, neuroticism and TCK towards TPACK. Using PLSpredict, the proposed model demonstrated substantial predictive power over the LM model, consistently achieving lower RMSE and MAE across most indicators. The students’ academic performance improved through the online course enrolled in the proposed C-LMS.

**Conclusions:**

The current research demonstrated the proposed structural model, adopting the Big Five Personality Traits (BFPT) and the TPACK framework, and integrating the proposed C-LMS to improve students’ collaborativeness through a dedicated online course. Moreover, students’ understanding of the enrolled online course improves significantly when the proposed C-LMS is integrated as the primary virtual collaborative learning environment. The phenomenon explained the positive impact of the proposed C-LMS on students’ academic performance in collaboration and knowledge co-creation.

## 1. Introduction

### 1.1 Overview

Technology is an inseparable element of the modern era, and its progressive evolution is inevitable. Over the decades, technology has been integrated into various industries, including education. Therefore, educational technology (EdTech) is a product of technological advancement. The effect of technology integration is amplified during the COVID-19 outbreak, as schools were closed, leaving a shocking impact on the educational community. As a result, the world is awash with countless technological solutions to address the new global trend ( Ravichandran et al., 2024). It catalyses the transition of the education sector into the digital era. As a result, demand for edtech has surged since then.

In higher education, the means of knowledge transfer and delivery have changed due to the adoption of edtech. The restrictions during the pandemic unlocked the potential of edtech, enabling a transition from the traditional classroom to the virtual classroom, with edtech serving as an assistive tool that supports teachers in the virtual environment ( Law et al., 2023; Lai et al., 2021). However, the practice of conventional teaching and learning can hardly be translated into virtual classroom settings. In the virtual environment, the interactions between teachers and students are hindered. Unlike in the conventional classroom, interactions and collaborations between teachers and students are challenging to occur virtually ( Salarvand et al., 2023). Besides, teachers’ technological proficiency is also a concern due to the swift transition from traditional to virtual classroom environments, leaving them with limited digital pedagogical experience to cope with the sudden change ( Lai et al., 2025; Teichert et al., 2023). Hence, concerns regarding teachers’ technology proficiency and the lack of interaction and collaboration between teachers and students arose due to the sudden shift in the teaching and learning environment.

Aside from the teaching and learning environment, students’ personality traits are among the factors that influence academic performance and can sometimes be overlooked. Personality traits play a vital role in education, shaping students’ levels of interaction and academic success ( Yildiz Durak, 2023). On top of that, although previous studies showed that the relationship between personality traits and academic performance was robust ( Lyons et al., 2017; Vasileva-Stojanovska et al., 2015), the topic required more findings as the educational trends change over time. The changes might affect the perspective on the relationship between personality traits and academic performance. Moreover, the rapid growth of virtual education during the pandemic opened more possibilities for conducting lessons. The scenario also indicates that education is no longer bound to fixed, or rather conventional methods, but is embracing various forms of technology integration to provide a more tailored and personalised environment for teaching and learning ( Rahman et al., 2022; Weiling et al., 2025). It is more pronounced in virtual education. Therefore, it is essential to consider personality traits in the present study to examine their relationship with academic performance in modern education.

Speaking of modern education, conventional teaching methods are no longer sufficient to fulfil the needs of teaching and learning in the new norm. Thence, the pedagogical content knowledge (PCK) framework introduced by Shulman in 1986 is unable to address the additional technology component commonly integrated into modern pedagogy ( Shulman, 1986). In 2006, Mishra and Koehler introduced the technological, pedagogical, and content knowledge (TPACK) framework to address the role of technology in modern education ( Mishra & Koehler, 2006). TPACK framework. Therefore, the TPACK framework consists of 3 domains: (1) technological knowledge (TK), (2) pedagogical knowledge (PK), (3) content knowledge (CK), and 4 sub-domains: (1) technological pedagogical knowledge (TPK), (2) technological content knowledge (TCK), pedagogical content knowledge (PCK), (4) technological pedagogical content knowledge (TPCK). It is a conceptual framework that emphasises the assessment of pre-service teachers’ efficacy in integrating technology into their teaching. A questionnaire was designed with questions dedicated to each domain and subdomain, allowing pre-service teachers to assess themselves through self-reporting. However, the self-reporting questionnaire design has underlying bias concerns. Previous studies indicated that the teacher’s TPACK self-assessment questionnaire may introduce bias, leading to either under- or overestimation of results ( Atmojo et al., 2025; Gonscherowski & Rott, 2025; Petridou et al., 2025; Su & Foulger, 2019). On top of that, students of modern education are involved in various technology integrations into their learning. Thus, it is vital to examine TPACK from their perspective, as their online learning experience is heavily dependent on their technology proficiency.

In light of the challenges outlined, the current research aims to further examine the stated issues by developing a framework that addresses students’ personality traits, continued use of technology, and collaborative learning in higher education.

### 1.2 Problem statements

Current educational research often focuses on a technology perspective, overlooking students’ personality traits. Their academic performance is often related to the teaching and learning environment, which is heavily integrated with pioneering technology. Oversight regarding how student personality traits influence academic performance can hinder the acquisition of valuable insights. Besides, the TPACK framework is well-known as a foundational framework for assessing pre-service teachers’ technology integration. However, previous research heavily favours the teachers’ perspective, overlooking that students are the primary users whose learning depends on technological integration. Furthermore, post-pandemic, academic institutions have shifted from physical to virtual classroom settings, mainly due to the educational continuity enabled by the virtual learning environment. Nonetheless, the conventional teaching practices and approaches are unlikely to be directly translated into the virtual environment without sacrificing the social components. Hence, research questions are drawn as follows:

- Research Question 1: How do higher education students’ personality traits influence their TPACK proficiency in a virtual collaborative learning environment?
- Research Question 2: How does higher education students’ TPACK proficiency influence their continued use of technology in a virtual collaborative learning environment?
- Research Question 3: What factors influence higher education students’ academic performance in a virtual collaborative learning environment?

### 1.3 Research objectives

The virtual classroom has become a common practice in modern education, and it is crucial to further investigate its potential in light of current educational trends. Consequently, a collaborative learning management system (C-LMS) is proposed and developed to cope with the current educational trend. Thus,

- To develop and validate a framework combining students’ personality traits, TPACK, and the continued use of technology
- To examine the relationship between higher education students’ personality traits and their academic performance within the virtual collaborative learning environments.
- To investigate TPACK from the student perspective and their continued use of technology.

## 2. Literature review

The shift from conventional to modern education settings depicts a pivotal turn in the literature. In the literature review subsections, the topics to be discussed are collaborative learning, learning management systems, personality traits, the TPACK framework, and their recent findings.

### 2.1 Collaborative learning

Collaborative learning is an umbrella term that encompasses a variety of educational approaches. It gained attention since the late 1950s, when research on the nature of collaboration emerged. In the past, students who engaged in collaborative learning were shown to outperform those who did not ( Bruffee, 1987). It highlighted the edge of collaborative learning among students; however, it also defied the traditional classroom autonomy where the teacher is the centre of classroom activities. In other words, the classroom activities were conducted solely in a teacher-centred fashion. Due to traditional classroom autonomy constraints, teachers play a vital role in designing assignments and ensuring that activities and tasks involve student-to-student interaction ( Arafat & Khshali, 2025). In addition, shifting teachers’ roles to facilitators shifted the focus of classroom activities from one-way lecturing to co-communication and mutual interaction among students. The teachers are responsible for structuring an environment that encourages student-to-student interaction while assisting them throughout the collaborative learning process ( Bjelobaba et al., 2023). It promotes lifelong learning rather than traditional content memorisation, which has been practised for decades. As such, the mode of autonomy shifted from teacher in full control to partial autonomy, in which teachers do not interfere with their students’ work after their group is formed, and the task is assigned. It introduced a different form of collaboration in the virtual learning environment, especially given the limited presence of teachers. There are uncertainties underlying the swift transition and adoption of collaborative learning in the virtual learning environment that are worth further studying in the current research.

Furthermore, rapid technological evolution has led to the trend of integrating technology into education, serving as a supportive role in teaching and learning. In the meantime, it also enhanced the collaborative learning experience through modern technologies, including physical hardware devices and peripherals, as well as virtual applications and software ( Holly et al., 2025). Therefore, collaborative learning is no longer limited to physical interactions between students, as technology enables a wide variety of new-era pedagogical approaches. Given the widespread use of computers nowadays, teachers and students have the luxury of choosing how classes are conducted. Besides, the means of achieving collaborative learning broaden with the introduction of computer-supported collaborative learning (CSCL) ( Adhami & Taghizadeh, 2024). CSCL is a process that utilises computers, software, and applications to determine their learning preferences. It shifts classroom autonomy from teacher-centered to student-centered, granting students control over their learning pace and co-creation of knowledge. It enriches their learning experience by offering various types of learning materials that pique their interest. Hence, collaborative learning offers students vast opportunities to further discover and assimilate information, especially with the help of a technological perspective, which is a worthwhile research direction to be further explored.

### 2.2 Learning Management System (LMS)

Before the utilisation of LMS in education, teachers and admin relied solely on pen and paper for recording academic matters and storing them in a cabinet filing system. Aside from its ineffectiveness in maintaining data integrity and consistency, it does not provide any insightful information. As a result, educational institutions often adopt LMSs, replacing the legacy file system that has been in place for decades. The transition from legacy file systems to LMS adoption changes the means of teaching and learning, as well as the acquisition and management of academic records ( AL-Nuaimi et al., 2024). According to Sulaiman et al. (2023), their study suggests that LMSs designed to be simple and accessible are often preferred by teachers, as they find them easy to use ( Sulaiman et al., 2023). Additionally, unlike previous studies, Sulaiman et al.’s study revealed that the LMS’s usefulness and ease of use were merely affected by the availability of technical support. It contradicts the claim that LMS with adequate support lowers the learning curve, thereby helping teachers perform desired tasks more easily. However, the study focused solely on teachers’ perspectives, overlooking students’ thoughts and opinions, even though they are also users of the LMS.

According to Dahal and Manandhar’s (2024) study, LMS is well-suited for managing resources and academic content ( Dahal & Manandhar, 2024). The LMS bridges technology and education, aligning with the modern education trend that treats technology as a core element in the presentation and delivery of knowledge. However, despite the remarkable benefits LMSs have offered, their study suggested that there is still room for improvement in the communicative features, engagement, and assessment. Those were the aspects that LMSs strive to outperform traditional classroom settings. Besides, according to Ahmad et al. (2023), limited computer literacy and limited support availability were among the major reasons teachers neglect to integrate and utilise LMS into their pedagogy. The issues are closely tied to the technology competency of teachers and students, as they are the primary users of LMSs. Thus, it is vital for current research to study the design and features of LMSs and identify their full potential, especially from an analytical perspective.

### 2.3 Technological integration framework with inclusion of personality traits in higher education

Within the vast field of education research, highlights often focus on pedagogical approaches, technology integration, and engagement. On top of that, the well-known TPACK framework was introduced by Mishra and Koehler in 2006 which is shown in Figure 1, focusing solely on assessing pre-service teachers’ efficacy in integrating technology into their teaching, which is insufficient to provide a clearer picture of current technology integration in education ( Gatete, 2025).

**Figure 1.**
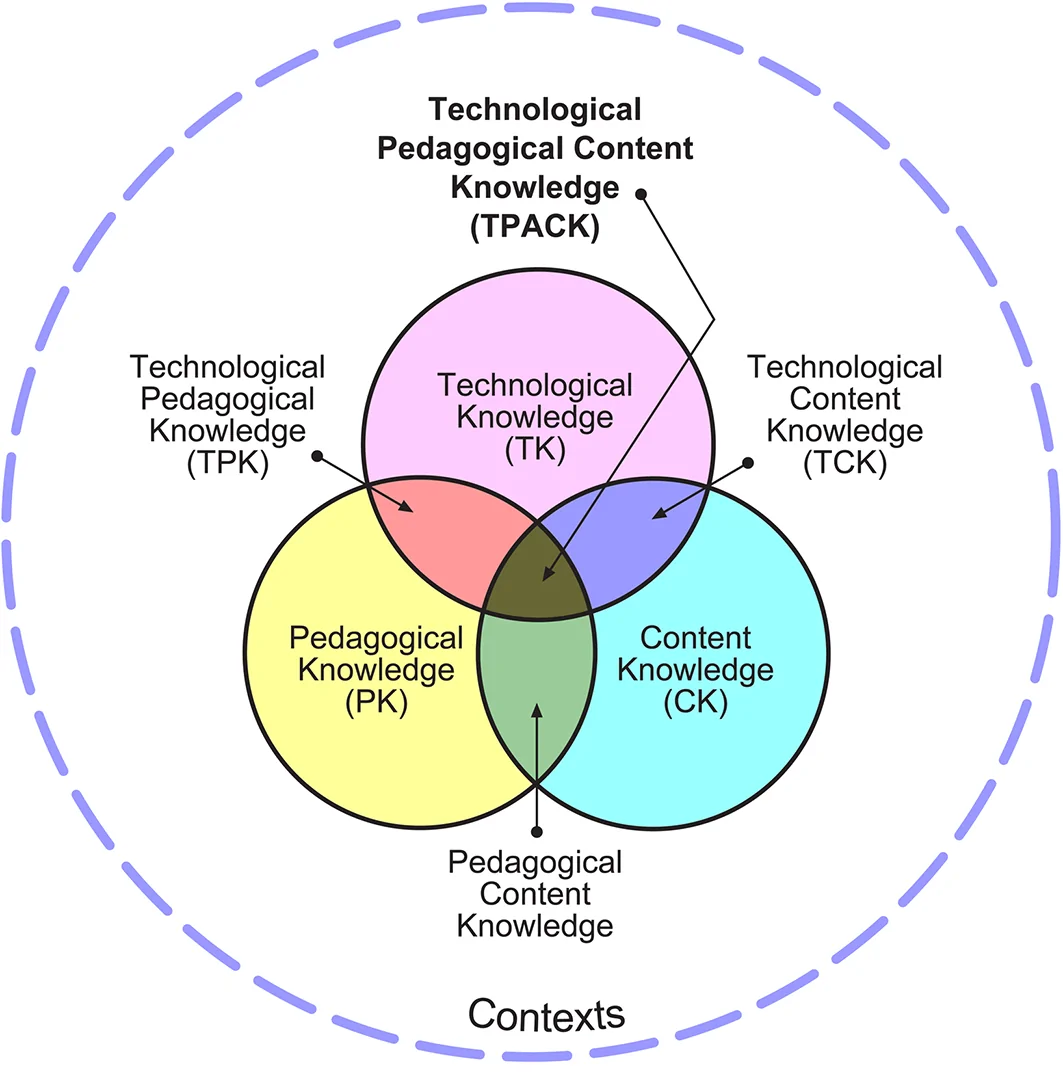
TPACK framework. An overview of the domains and subdomains of the TPACK framework by Koehler and Mishra.

As a result, students’ personality traits are generally overlooked, though they offer valuable insights for teachers to assess their students from different perspectives. In terms of personality traits, the Myers-Briggs Type Indicator (MBTI) and the Big Five Personality Traits (BFPT), also known as the Five-Factor Model, are among the most widely known measures, which are shown in Figure 2 and Figure 3, respectively.

**Figure 2.**
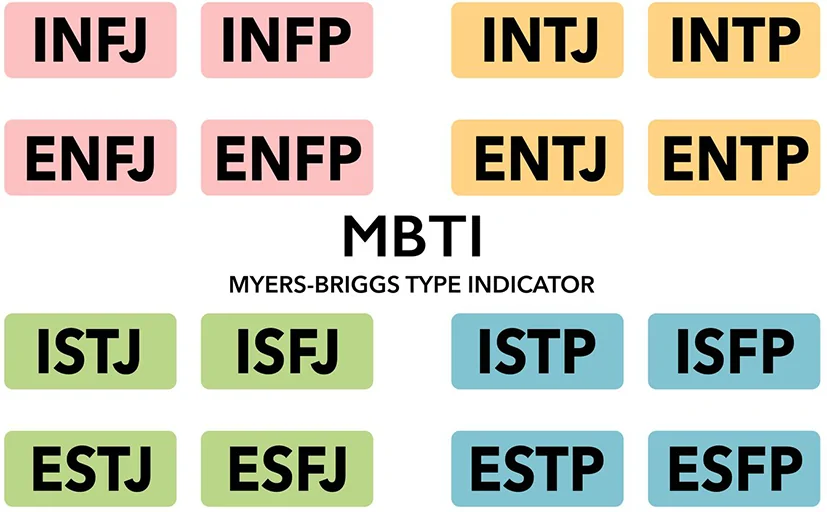
MBTI. An overview of the MBTI, which was introduced by Katharine Cook Briggs and Isabel Briggs Myers.

**Figure 3.**
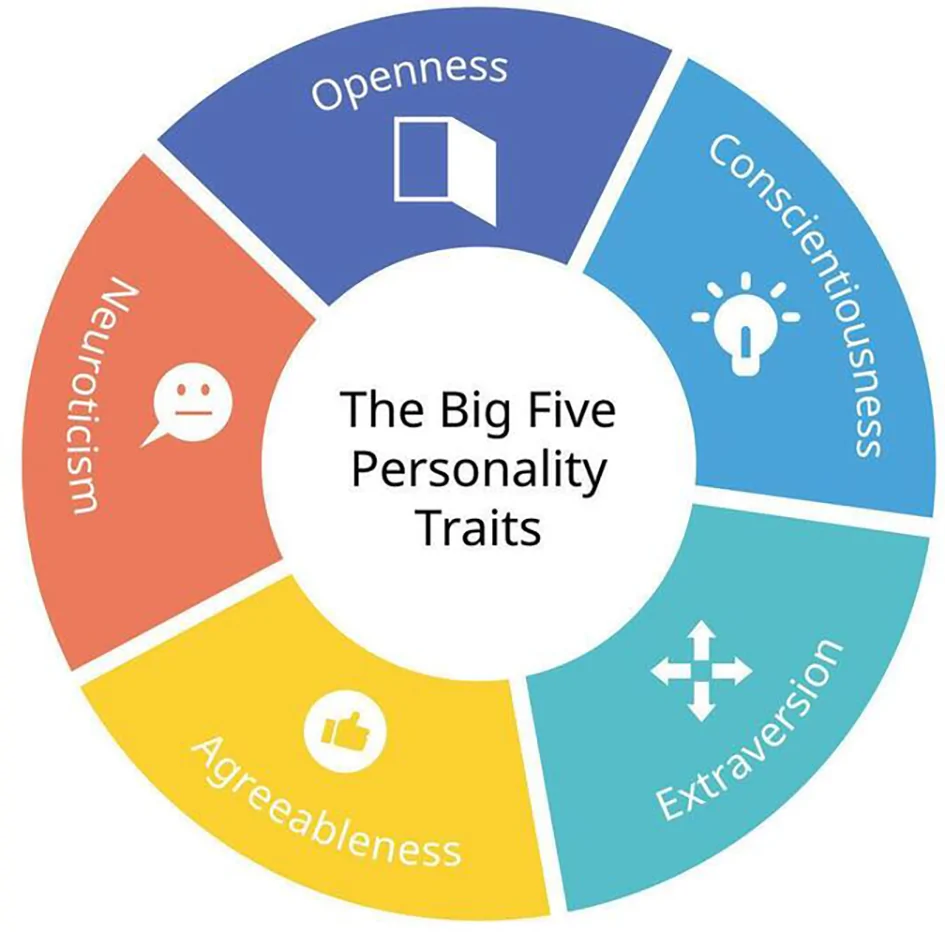
Big five personality traits. An overview of the big five personality traits, which were introduced by a cumulative research of multiple psychologists. Its origin is based on the five factors identified by D.W. Fiske (1949).

In recent years, MBTI has become popular since the end of 2019. This phenomenon is more noticeable in Asia than in its origin, the United States. Although corporations adopt it as a marketing tool when seeking the young talent they desire, it remains a pseudoscience whose fundamental principles are incompatible with the scientific method ( Ma, 2025). In other words, MBTI lacks empirical evidence, leading to confirmation bias and vague claims. With due reason, MBTI does not appeal to the scientific community because of its lack of scientific validity.

Unlike MBTI, which provides a narrative framework specifically for self-reflection, BFPT is more psychometrically stable, making it an ideal option for predictive social science research. The BFPT was used as the predictor of student academic performance. It is mainly due to its high reliability and statistical validity compared to MBTI ( Shaninah & Mohd Noor, 2024; H. Wang et al., 2023). Moreover, each of the five dimensions of BFPT explicitly describes a person’s personality, which is often conceived as more independent and stable, and the model is psychologically grounded in this conception compared to MBTI. From an educational perspective, past studies have suggested that BFPT is closely related to educators’ effectiveness in their profession from various aspects, such as teaching performance and mental exhaustion level ( Liu et al., 2022). As a result, the BFPT of oneself can affect the level of collaboration and the educational experience within the academic institution. Henceforth, the BFPT is further studied in current research from students’ perspectives, yielding valuable insights into the relationship between BFPT and students’ academic performance as well as continued use of technology.

To further explain the relationship between students’ BFPT and the technology integration framework, the current research extends the BFPT by integrating it with the technology integration framework. Although models such as the Technology Acceptance Model (TAM) and the Unified Theory of Acceptance and Use of Technology (UTAUT) emphasise technology and its utility and features rather than pedagogical approaches ( El Alfy & Kehal, 2024; Lotey et al., 2023), they do not address pedagogical approaches. Therefore, the current research adopted the TPACK framework, as the additional technological knowledge domain enables it to fit within modern education. In previous studies, TPACK has been used to assess pre-service teachers’ self-efficacy in integrating technology into their teaching ( Bwalya & Rutegwa, 2023; Joshi, 2023; Thohir et al., 2023). However, these studies were solely focused on teachers’ perspectives, with results collected through self-assessment using a dedicated TPACK questionnaire. The classroom activities mainly involve two parties: teachers and students. It is essential to acquire input from students’ perspectives because it is as valuable as input from teachers’ perspectives, as the teachers’ efficacy directly affects the students’ learning experience. In the virtual environment, teachers and students are both users of technology. Hence, the current research examines TPACK from the students’ perspective, with BFPT as the predictor in the research framework.

## 3. Methods

### 3.1 Research framework

The proposed research framework in the current research is shown in Figure 4. The research framework is developed with the proposed collaborative learning management system (C-LMS) as the primary environment for learning activities. The Big Five Personality Traits (BFPT) serve as the exogenous predictors that comprise: (1) openness to experience, (2) conscientiousness, (3) extraversion, (4) agreeableness, and (5) neuroticism. Besides, the TPACK domains and subdomains considerations in the current research are solely focusing on the technology-related ones, namely: (1) technological knowledge, (2) technological content knowledge, (3) technological pedagogical knowledge, and (4) technological pedagogical and content knowledge. The dependent variable in this research is continued use of technology.

**Figure 4.**
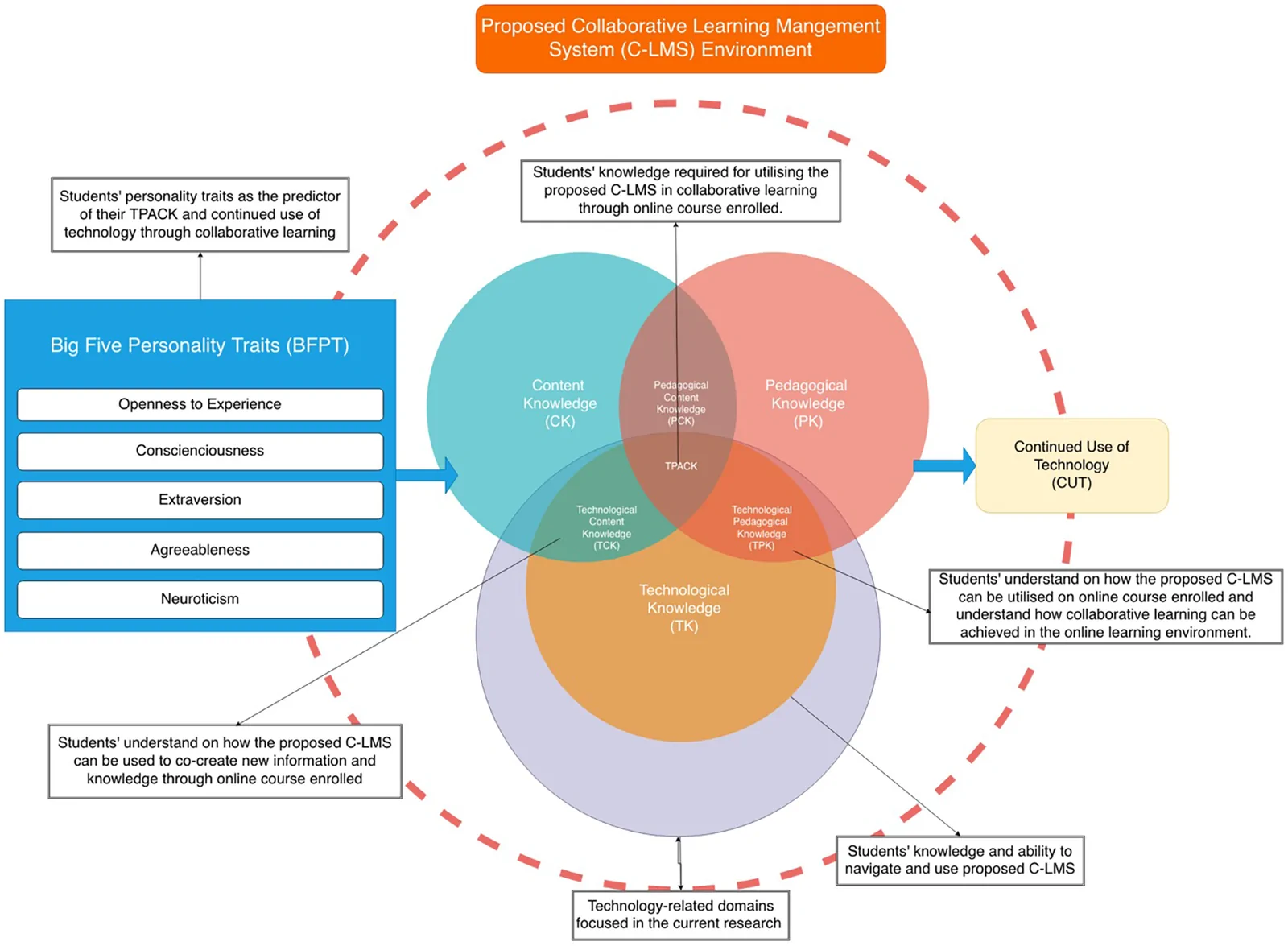
Proposed research framework. An overview of the proposed research framework of the current research. It highlighted the adoption of the TPACK framework in the context of the proposed C-LMS as the primary learning environment. It also highlighted the inclusion of BFPT in forming the research framework.

### 3.2 Formulate hypotheses

The BFPT-TPACK framework is formulated in the current research, as shown in Figure 5. The framework adopts the BPFT and TPACK framework with an additional variable that inspects the influences on students’ continued use of technology. On top of that, the current research focuses on the technology-integrated collaborative learning environment, which explains the inclusion of only the technology-related domains and subdomains of the TPACK framework ( Johnson et al., 2024). Henceforth, this research aims to establish a more in-depth understanding of the interconnection between the BFPT and TPACK, as well as of students’ continued use of technology from their perspective.

**Figure 5.**
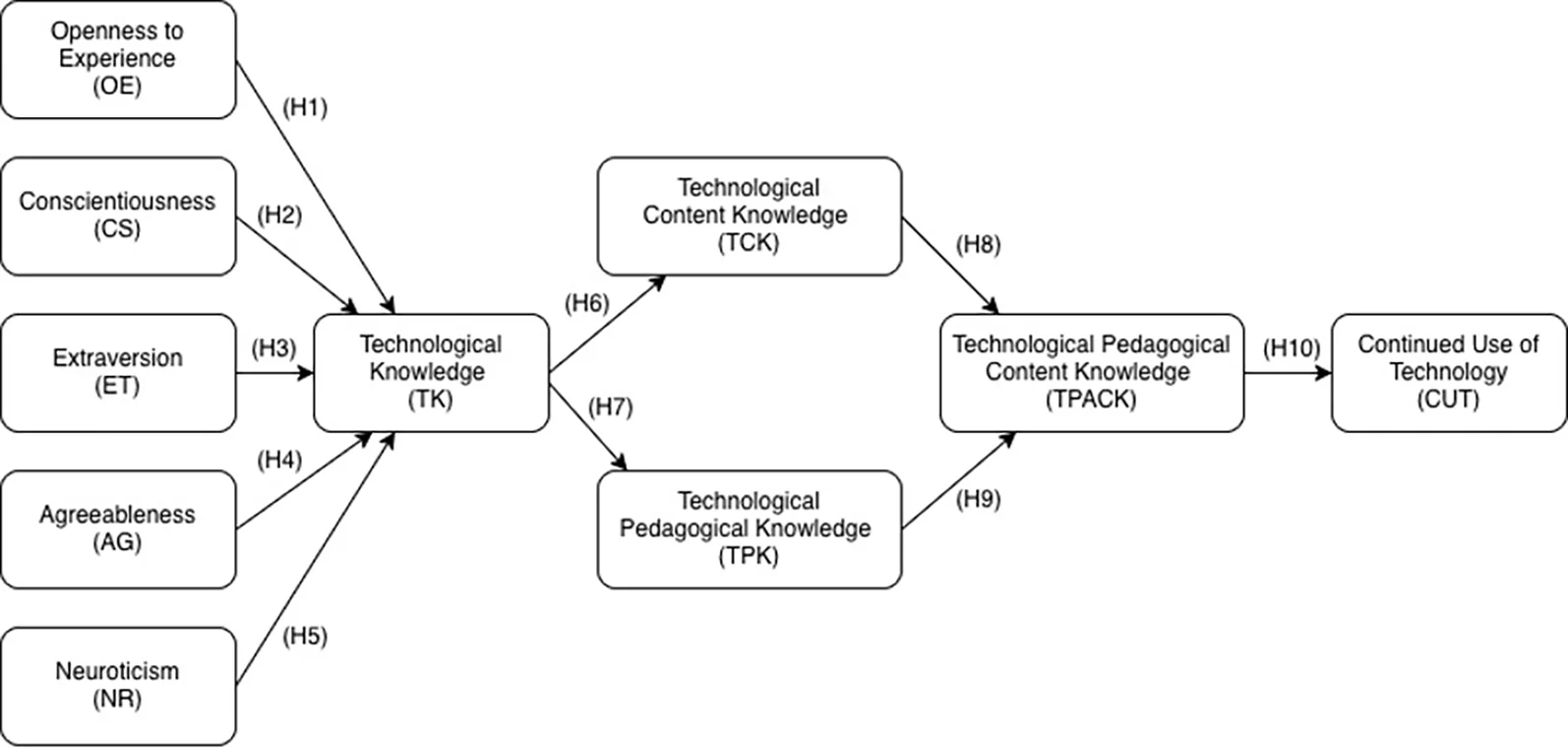
BFPT-TPACK framework. An overview of the BFPT-TPACK framework with hypotheses labeled from H1 to H10.

The hypotheses of the research are as follows:

H1: OE predicts TK positively in the virtual collaborative learning environment.

H2: CS predicts TK positively in the virtual collaborative learning environment.

H3: ET predicts TK positively in the virtual collaborative learning environment.

H4: AG predicts TK positively in the virtual collaborative learning environment.

H5: NR predicts TK positively in the virtual collaborative learning environment.

H6: TK predicts TCK positively in the virtual collaborative learning environment.

H7: TK predicts TPK positively in the virtual collaborative learning environment.

H8: TCK predicts TPACK positively in the virtual collaborative learning environment.

H9: TPK predicts TPACK positively in the virtual collaborative learning environment.

H10: TPACK predicts CUT positively in the virtual collaborative learning environment.

### 3.3 Sampling technique

In general, sampling techniques can be categorised into two major types, namely: (1) probability sampling, and (2) non-probability sampling ( Farid Shamsudin et al., 2024). Non-probability convenience sampling was used in the current research, where the participants were assigned to different tutorial groups before the experiment began. The research involved 121 local private university students enrolled in the online course via the proposed C-LMS.

### 3.4 Overall experiment flow

The proposed C-LMS is a site designed and developed with features that meet modern educational needs, specifically customised for the virtual learning environment. On the site, a dedicated online course is created and tailored for higher education students to foster collaborative learning in a virtual environment, with in-depth analytical insights. The existing LMS lacks the capability to support collaborative learning, which aligns with modern educational needs ( Hemphill et al., 2023; Jaikrishin Belani et al., 2025; Saadati et al., 2023). Hence, the current research proposed a more direct approach to an online course with group-based activities such as group assignments, an open forum, and a glossary. These activities encourage students to interact with their peers and groupmates. The proposed C-LMS is designed to be a cross-platform web application that allows teachers and students to access the site on personal computers, smartphones, and tablets without distorting the user interface across platforms. The proposed C-LMS supports synchronous and asynchronous learning, accommodating students’ preferences for time management. It allows the students to access the learning materials and learn at their own pace. In the meantime, they can interact with their teachers and peers through dedicated spaces, such as real-time chat and forums, to share their thoughts and foster collaboration.

During the experiment phase, a group of local higher education students enrolled in the dedicated online course through the proposed C-LMS. The online course consists of three modules and a post-test assessment as the closure. The students are divided into small groups of 4. Before the students embark on their journey to explore and commit to the online course learning materials, they are required to complete a survey questionnaire about their BFPT. The questionnaire adopted the Big Five Inventory-20 (BFI-20), consisting of 20 self-reported items, with the 4 best-performing items for each personality trait selected from the 44-item Big Five Inventory (BFI-44). After completing the questionnaire, the students are open to accessing real-time chat, open discussion forums, and the learning materials of the first module. Students can proceed to the next module only after completing the first module. Each module offers students video and reading materials to choose from. Students must complete a pre-module knowledge-checking test before they are granted access to the module’s learning materials. The students are then required to work with their groupmate to create a glossary of what they have learnt from that particular module. The same steps are repeated for the remaining modules in the online course. Once all modules are completed, they are required to work in a group on a post-module course reflection, reflecting on what they have learnt throughout the course. After submitting the post-module course reflection, students need to complete a modified TPACK questionnaire. Upon submitting the modified TPACK questionnaire, students are considered to have completed the online course on the proposed C-LMS.

## 4. Results

In the current research, the proposed model is constructed through PLS-SEM using SmartPLS 4.0 as a reflective measurement model. The proposed model is shown in Figure 6.

**Figure 6.**
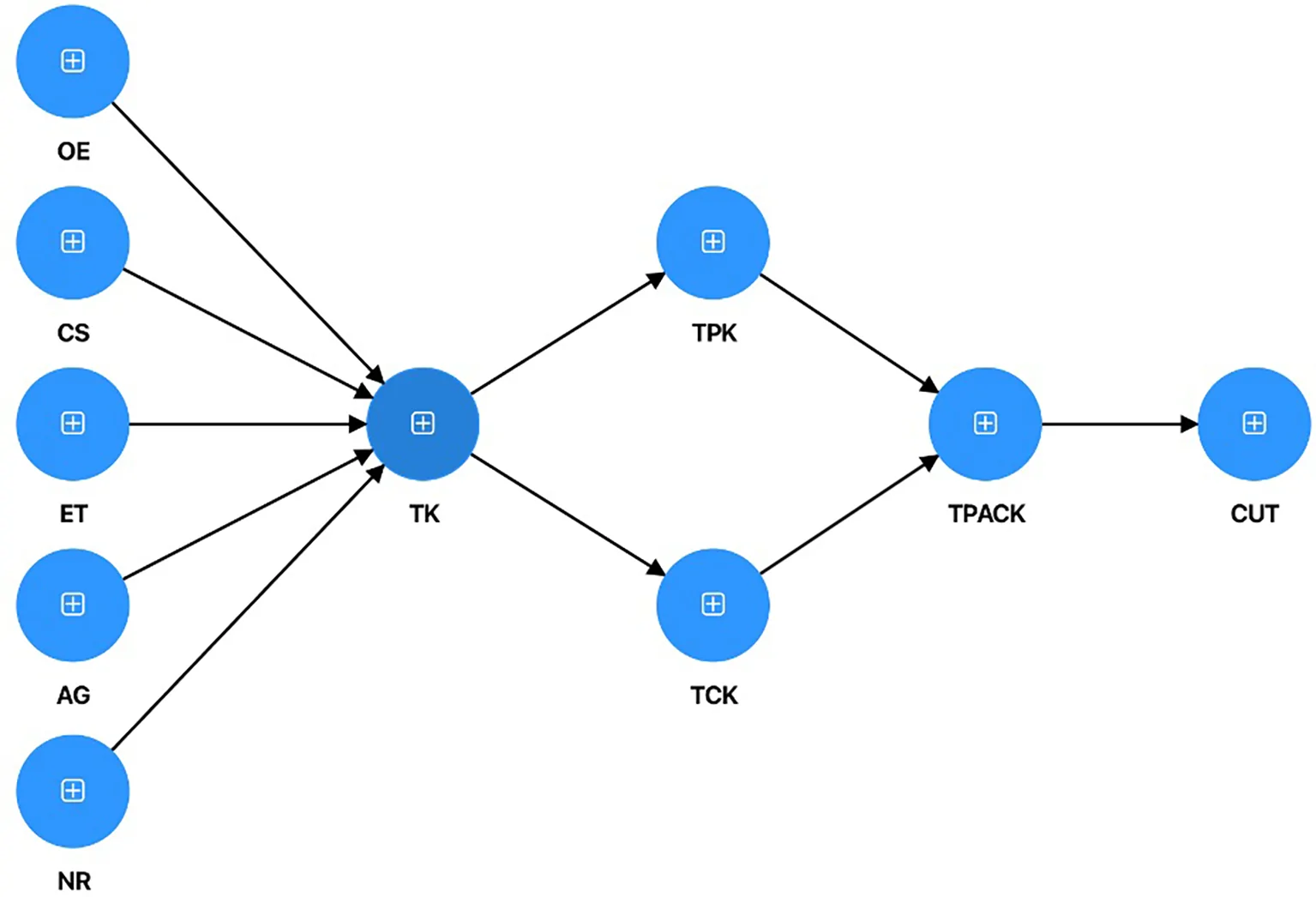
Proposed research model. An overview of the proposed research model, including BFPT, technology-related TPACK domains and subdomains, and continued use of technology, is presented.

Upon running the Partial Least Squares (PLS) algorithm, the constructs’ outer loadings indicators are shown in Supplementary Table 1, with construct indicators loading > 0.7, except AG2 with the value 0.664. AG2 is not removed because the AG met the construct reliability, validity, and discriminant validity requirements, despite a loading of < 0.7.

Next, the reliability and validity of the constructs are presented in Table 1. The composite reliability (CR), also known as Jöreskog ρ, has a threshold value of > 0.7 ( Nallaluthan et al., 2024). In a recent study, DiJkstra-Henseler’s rho (ρA) was considered a more precise measure of construct reliability than conventional composite reliability (ρC), with a threshold of > 0.7 ( Dijkstra & Henseler, 2015; B. Wang et al., 2026). In addition, the average variance extracted (AVE) value threshold is > 0.5. In the current research, the CR and AVE fulfilled the threshold requirements with CR (ρC) values > 0.75 and AVE values > 0.50. The values of CR and AVE indicate good reliability and validity, respectively.

**Table 1.** Constructs’ reliability and validity.

Construct(S)	Cronbach’s alpha	Composite reliability (rho_a)	Composite reliability (rho_c)	Average variance extracted (AVE)
AG	0.851	0.917	0.899	0.692
CS	0.902	0.905	0.931	0.772
CUT	0.861	0.870	0.915	0.781
ET	0.885	0.930	0.917	0.734
NR	0.917	0.922	0.941	0.800
OE	0.886	1.000	0.915	0.729
TCK	0.904	0.919	0.939	0.838
TK	0.837	0.836	0.903	0.756
TPACK	0.757	0.808	0.855	0.663
TPK	0.851	0.917	0.899	0.692

*Note*: The table indicates the constructs’ reliability and validity of the current research. It consists of cronbach’s alpha, composite reliability (RHO_A), composite reliability (RHO_C) and average variance extracted (AVE) of the proposed research model.

Furthermore, the model’s discriminant validity is assessed through Heterotrait-Monotrait Ratio (HTMT) and shown in Table 2. It is ideal as it has stringent measures with 97-99% sensitivity rates ( Ab Hamid et al., 2017). Unlike HTMT, the Fornell-Larcker Criterion is comparatively insensitive, with a value of 20.82%. The HTMT values in the current research met the HTMT threshold, with values generally < 0.85. Therefore, the discriminant validity between relative constructs is achieved.

**Table 2.** Heterotrait-Monotrait Ratio (HTMT) for discriminant validity.

	AG	CS	CUT	ET	NR	OE	TCK	TK	TPACK	TPK
AG										
CS	0.136									
CUT	0.348	0.093								
ET	0.601	0.404	0.177							
NR	0.351	0.069	0.310	0.076						
OE	0.786	0.260	0.139	0.860	0.112					
TCK	0.186	0.765	0.177	0.173	0.053	0.068				
TK	0.393	0.697	0.129	0.148	0.219	0.095	0.828			
TPACK	0.512	0.243	0.420	0.169	0.278	0.169	0.354	0.585		
TPK	0.342	0.328	0.380	0.294	0.193	0.198	0.449	0.447	0.735	

*Note*: The table indicates the heterotrait-monotrait Ratio (HTMT) for the discriminant validity of the proposed research model.

The significance and relevance of the structural model are examined and presented in Table 3. The results are obtained using a Two-Tailed test and a bias-corrected and accelerated bootstrap (BCA) with bias and skewness adjustment. Since the research hypotheses are directional, the current research executes bootstrapping with 8000 subsamples. The path coefficients are then obtained for further examination, with the path coefficient values at least reaching the 0.05 significance level.

**Table 3.** Path coefficient.

Path	Original sample (O)	Sample mean (M)	Standard deviation (STDEV)	T statistics (|O/STDEV|)	P values	Result
AG - > TK	0.626	0.573	0.139	4.505	0.000***	Significant
CS - > TK	0.705	0.690	0.080	8.777	0.000***	Significant
ET - > TK	−0.168	−0.169	0.113	1.480	0.139	Insignificant
NR - > TK	−0.021	−0.008	0.067	0.309	0.757	Insignificant
OE - > TK	−0.373	−0.308	0.183	2.043	0.041*	Significant
TCK - > TPACK	0.106	0.108	0.076	1.402	0.161	Insignificant
TK - > TCK	0.731	0.729	0.066	11.003	0.000***	Significant
TK - > TPK	0.370	0.373	0.086	4.281	0.000***	Significant
TPACK - > CUT	0.374	0.385	0.086	4.369	0.000***	Significant
TPK - > TPACK	0.565	0.571	0.065	8.690	0.000***	Significant

*Note*: The table indicates the significance of the path coefficient of the proposed research model.

Moreover, the R ^2^ and R ^2^ adjusted values are presented in Table 4. It is intended to determine the predictive accuracy of the structural model. The R ^2^_adj_ values for the CUT, TCK, TPACK, and TPK are considered to have substantial predictive power ( Cohen, 1988; Putu Gede Subhaktiyasa, 2024). Conversely, the CUT and TPK R ^2^_adj_ values are 0.132 and 0.130, respectively, indicating low predictive power.

**Table 4.** R ^2^ and R ^2^ adjusted values.

	R squared	R Squared adjusted
CUT	0.140	0.132
TCK	0.535	0.531
TK	0.589	0.571
TPACK	0.374	0.364
TPK	0.137	0.130

*Note*: The table indicates the R ^2^ and R ^2^ adjusted values of the proposed research model.

Additionally, the effect sizes of the framework’s exogenous latent variables were determined using the f ^2^ statistic. It evaluates how strongly an exogenous latent variable contributed to explaining the endogenous latent variable. The effect size is categorised into: (1) a very small effect size of f ^2^ values < 0.02, (2) a small effect size of f ^2^ values in the range of 0.02 – 0.14, (3) a moderate effect size of f ^2^ values in the range of 0.15 – 0.34, and (4) a large effect size of f ^2^ values > 0.35 ( Cohen, 1992; López Martín & Ardura Martínez, 2023). Moreover, the f ^2^ value can be calculated using Equation 1. In the meantime, the f ^2^ results are demonstrated in Table 5.

$$f^{2}=\frac{R^{2}}{1-R^{2}}$$

**Table 5.** F ^2^ results.

Path	Original sample (O)
AG - > TK	0.459
CS - > TK	1.021
ET - > TK	0.029
NR - > TK	0.001
OE - > TK	0.120
TCK - > TPACK	0.016
TK - > TCK	1.148
TK - > TPK	0.159
TPACK - > CUT	0.162
TPK - > TPACK	0.440

*Note*: The table indicates the f ^2^ result of the proposed research model.

Equation 1: f ^2^ formula

Therefore, from the BFPT perspective, ET and NR had negligible effects on TK, with f ^2^ values of 0.029 and 0.001, respectively, which fall within the very small effect size category. In terms of CS and AG, they substantially affect TK, with f ^2^ values of 0.459 and 1.021, respectively, which fall within the small effect size category. In terms of OE, it has a minor effect on TK with f ^2^ values of 0.120, which fall within the small effect size category. From the TPACK perspective, TCK had a slight effect on TPACK with an f ^2^ value of 0.016, which falls within the very small effect size category. As for TPK, it had a significant effect on TPACK with an f ^2^ value of 0.440, which falls within the large effect size category. As for TK, it substantially affects TCK and moderately affects TPK, with f ^2^ values of 1.148 and 0.159, respectively, which fall within the large and moderate effect size categories, respectively. Lastly, TPACK had a substantial effect on CUT with an f ^2^ value of 0.162, which falls within the moderate effect size category.

Furthermore, the results of the Manifest Variables (MV) prediction summary conducted with PLSpredict are presented in Supplementary Table 2. It is used to evaluate the out-of-sample predictive capabilities of PLS path models ( Shmueli et al., 2019). It has 3 steps when performing predictive relevance through the PLSpredict algorithm: (1) check Q ^2^predict, (2) compare PLS-SEM against the LM errors, and (3) classify the predictive power. In the first step, the Q ^2^predict values of every indicator for TK, TCK, TPK, TPACK, and CUT are > 0, which proves the model outperforms the most naïve baseline. Thus, step 2 is proceeded. In the second step, the proposed model’s error is compared with the Linear Model (LM) benchmark for each indicator: TK, TCK, TPK, TPACK, and CUT. The PLS-SEM model produced lower prediction errors (RMSE) than the LM benchmark, as evidenced by the difference between the proposed model’s RMSE and the LM benchmark’s RMSE for most indicators. In the third step, the predictive power of the proposed model is determined by the number of indicators that achieved lower prediction errors (RMSE) than the LM model. The proposed model has lower prediction errors in RMSE and MAE than the LM model across as the majority of the model indicators, which are 11 out of 15 indicators.

The results from the structural framework assessment are illustrated in Figure 7. The ET and NR from BFPT were removed from the framework due to their insignificant predictive effect on the TPACK element, namely TK.

**Figure 7.**
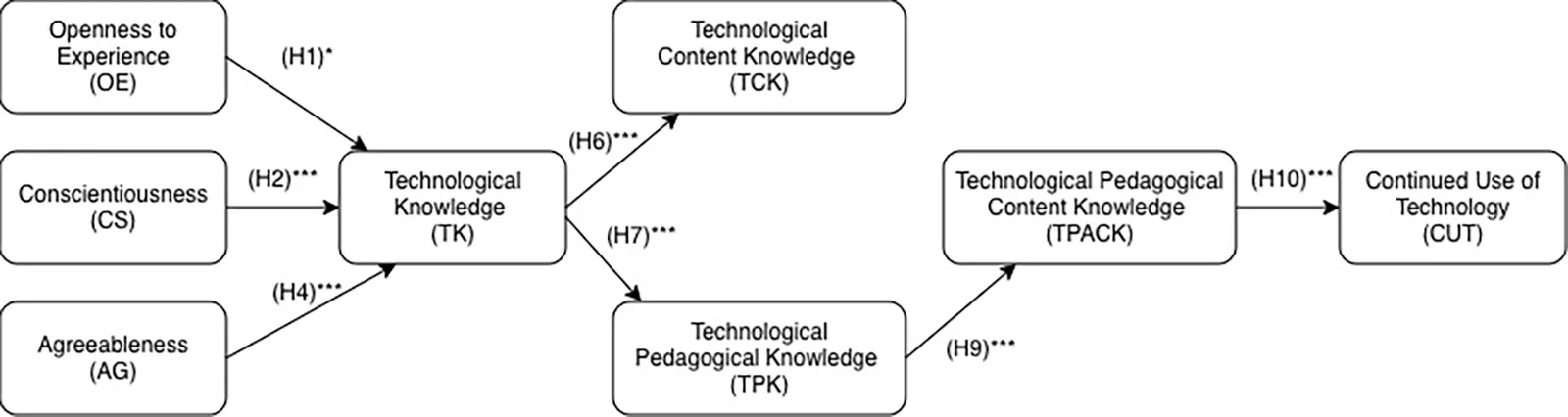
BFPT-TPACK Framework after Measurement and Structural Analysis. Remark: *p < 0.05, **p < 0.01, ***p < 0.001. The line was removed because the p-value was insignificant. An overview of the BFPT-TPACK framework’s significance after measurement and structural analysis.

A paired-samples T-test was performed as shown in Table 6. It was meant to examine students’ improvement in their understanding of the module content of the enrolled online course through the proposed C-LMS. The improvement in the students was presented as a mean score difference of -4.88. The p-value < 0.001 indicates that the enrolled online course via the proposed C-LMS positively affects students’ academic performance, as the post-module knowledge-checking test (P_AVG) outperformed the pre-module knowledge-checking test (M_AVG), indicating a substantial improvement.

**Table 6.** Paired-Samples T-Test. The table shows the difference in academic performance between the students’ pre- and post-module knowledge-checking test.

			Statistic	df	p	Mean difference	SE difference
**M_AVG**	**P_AVG**	**Student’s t**	−25.8	120	<.001	−4.88	0.189

*Note*: H _a_ μ _Measure 1 - Measure 2_ < 0.

## 5. Discussion

The swift evolution of technology opens vast opportunities, allowing new ideas and solutions to bloom. The phenomenon accelerates the progression and advancement of educational technology. Given the prominent role of technology in current educational trends, the current research adopted the TPACK framework. Conventionally, TPACK has been used to assess pre-service teachers’ proficiency in integrating technology into their teaching approaches and materials. In modern education, educational technology such as computers, laptops, and learning management systems is inseparable from the students. Studying their perspectives on the technology is valuable as they are the primary users. However, the students’ technological proficiency is not the sole factor affecting their online learning experience and habits. Considering the students’ psychological perspective, the current research adopted BFPT, as their personality traits reflect their actions and behavior in the virtual learning environment. Thence, the proposed model is constructed based on the adoption of TPACK and BFPT. The current research focuses on students’ perspectives as their roles change in the online learning environment. The proposed dedicated learning management system is designed to encourage collaborative learning amongst students. They carry out most of the activities in the online course with their peers and groupmates, while their teachers facilitate and assist them as needed. Therefore, student feedback is crucial for gaining in-depth insights into their traits in the virtual learning environment, allowing teachers to have an overview of how their personality traits affect their online learning behaviour and academic performance. From the path coefficient results obtained with SmartPLS4, we observe that students’ BFPT contributed to explaining their technological proficiency in the virtual learning environment and indirectly reflected their continued use of technology. A dedicated platform designed for modern learning approaches has the potential to enhance their academic performance. According to the paired-samples t-test, the mean difference between the pre- and post-module knowledge-checking test indicates a significant improvement in students’ knowledge co-creation and acquisition. Moreover, the current research demonstrates that students’ personality traits are closely related to their technology-related TPACK domains and to their continued use of technology in the proposed C-LMS, which serves as the virtual collaborative learning environment.

Besides, the proposed C-LMS is developed with collaborative-oriented activities, such as open forums, glossary, and group assignments. The students were offered an alternative learning process and experience that differed from conventional classroom settings. Since the proposed C-LMS is designed to be a student-centered, collaborative learning platform, student feedback is essential for assessing their proficiency and determining their continued use of the platform for online learning. In addition, the BFPT-TPACK framework comprises 10 hypotheses that investigate students’ proficiency in conducting collaborative learning in the online course of the proposed C-LMS. The hypotheses and summary of the findings acquired from the experiment are shown in Supplementary Table 3.

In the current research, the students’ personality traits: (1) OE, (2) CS, and (3) AG, positively affect their TK, in which H1, H2, and H4 were supported. The phenomenon explained that the OE trait contributes to students’ technological exploratory tendencies, leading them to proactively discover new software without prior or formal training. In terms of the CS trait, it contributes to students’ ability to persistently seek solutions when encountering technical glitches or errors. They also have a deeper understanding of the system’s core functions. As for AG, it portrays the students as most likely to learn technology through social interaction, which is achievable through the proposed C-LMS online course. The students might assist their peers and groupmates by providing informal technical support to help them navigate and progress through the online course.

Unlike these traits, NR and ET did not achieve statistical significance in predicting TK, in which H3 and H5 were rejected. The NR primarily measures students’ emotional stability and their tendency to experience negative emotions, such as self-doubt or anxiety. Although it has a massive impact on how students feel regarding technology, it hardly reflects their understanding of it. On top of that, NR is an effective trait that is strongly tied to an emotional perspective. It is different from TK, which is a cognitive construct. One can possess decent technical skills to navigate the proposed C-LMS but still be highly anxious about using the new system. As for ET, it often refers to one’s focus on sociability and actively seeking stimulation from the outside world. It did not achieve statistical significance as it merely reflects the students’ technology literacy. Moreover, students with high ET used the social features of the proposed C-LMS to gain TK, as did those with low ET or who are introverted, who gained TK through tinkering. These two opposing traits may have reached similar levels of technological proficiency, leading to a non-significant path to TK.

Furthermore, the TK serves as a prerequisite, demonstrating students’ technology literacy and proficiency with the proposed C-LMS. The results of the current research showed that as students’ technological proficiency increases, their ability to transform content based on it increases, indicating that TK -> TCK is statistically significant and supporting H6. It is also known as their perception of the technology’s epistemic affordances, where the catalyst for the relationship between technology and content lies. Additionally, the statistical significance of TK -> TPK indicated that the students understand and know how to learn with the proposed C-LMS, thereby supporting H7. In the current research context, the students understood the pedagogical affordances of technology and the knowledge co-creation that took place. In other words, the relevance of TK to TPK indicates that students’ effectiveness with the collaborative learning opportunities depends on the technical mechanics of the proposed C-LMS. Moreover, TCK is a statistically insignificant predictor of TPACK in the current research, rejecting H8. It suggests that if a student is part of a collaborative group, they often rely on their ‘tech-savvy’ peers in technological content integration. Thus, the students’ TCK does not directly influence their personal perceived “TPACK” as the group manages the complexity. In other words, a student’s TCK is no longer a significant predictor of their personal TPACK confidence in a virtual collaborative learning environment because the collaborative nature compensates for individual gaps. TPK is a statistically significant predictor of TPACK in the current research, supporting H9. It is most often treated as a critical predictor, especially in a collaborative classroom setting. It also reflects that the students understand the utility of the proposed C-LMS for collaboration. In other words, they understand how to learn with their peers through the proposed C-LMS as the primary educational technology. Hence, the students possess a significantly more developed TPACK in the current research.

Lastly, the path from TPACK to CUT is statistically significant, supporting H10 and providing empirical evidence that the long-term adoption of the platform is primarily influenced by students’ technology efficacy. The result suggested that the students are more likely to continue using the proposed C-LMS as they become more competent with it. Also, TPACK proficiency correlates with students’ user experience with the proposed C-LMS. High TPACK proficiency ensures students have a smoother user experience, which, in turn, serves as an antidote to technology abandonment. While the learning curve is often treated as a barrier to technology acceptance, students with high TPACK proficiency tend to be more focused on their learning goals, as they are proficient at navigating and utilising technology, thereby leading in continued use of technology in the future. In other words, the students’ continued use of technology depends on whether it helps them master the subject matter and on peer collaboration.

### 5.1 Implication of the study

In terms of theoretical implications, the current research extends existing TPACK studies by incorporating BFPT, thereby validating students’ continued use of technology in the virtual collaborative learning environment. The previous studies showed that technology integration outcomes were not as promising; in fact, technology was mostly blamed. Although the TPACK framework has been shown to be highly relevant to modern education, it focuses solely on teachers’ understanding and proficiency in integrating technology into their teaching. Nevertheless, studies on the TPACK framework from students’ perspectives are limited. There are even fewer studies investigating students’ personality traits when the TPACK framework is adopted as the fundamental framework. Thence, the current research has taken the initiative to enrich the literature, indicating that the BFPT positively influenced the students’ TK, leading to a positive impact on the technology-related TPACK domains and subdomains, explaining that the continued use of technology is highly relevant to the students’ TPACK proficiency in the virtual collaborative learning environment. Furthermore, the current research’s findings contributed to the existing literature by addressing how the virtual collaborative learning environment enhanced students’ academic performance. The results also showed that the conventional beliefs and concepts in education, such as pedagogy and teachers’ autonomy, are not applicable in the virtual collaborative learning environment. Thus, the current research can be considered an early adopter of extending and assessing the relationship between the TPACK framework and BFPT from the student perspective, with limited literature.

### 5.2 Limitations and recommendations

Several limitations were considered in the current research. One of the most substantial limitations is the number of respondents involved in the research experiment. It might not be able to represent a more diverse demographic of respondents. A larger sample size is recommended in future studies for greater accuracy and reliability. Furthermore, the research experiment was conducted solely through the proposed C-LMS, a virtual collaborative learning environment. Hence, the findings of the current research may not directly reflect the physical collaborative learning environment, especially their BFPT relationship with TPACK. The students’ attitude towards their continued use of technology may differ as well. Moreover, the research focused solely on developing one dedicated system to encourage collaborative learning among students. Thence, it is very encouraging for developing a variety of systems with different classroom settings and activities for future studies. Hence, the collected findings and results offer greater value and insights for future educational technology development.

## Ethical considerations

This study involving human participants was conducted in accordance with the ethical guidelines set forth by the University, as approved by the Technology Transfer Office of Multimedia University (Ethical Approval Number: EA0742025). Written informed consent was obtained from all participants. Personal data was kept confidential and were used solely for the purposes of this study. All participants were over 18 years of age; no minors were involved in the study.

## Generative AI statement

The authors declare that no Gen AI was used in the creation of this manuscript.

## Publisher’s note

All claims expressed in this article are solely those of the authors and do not necessarily represent those of their affiliated organizations, or those of the publisher, the editors and the reviewers. Any product that may be evaluated in this article, or claim that may be made by its manufacturer, is not guaranteed or endorsed by the publisher.

## Data Availability

Zenodo: BFPT-TPACK-C-LMS. https://doi.org/10.5281/zenodo.20395339. ( Lai, 2026a) This project contains the following underlying data:
• AcademicPerformancePairedTTest.xlsx (Comparing the academic performance of two groups post-experiment anonymously) • Dataset.xlsx (Anonymised answers to questionnaire, 5 – strongly agree, 4 – slightly agree, 3 – neither, 2 – slightly disagree, 1 – strongly disagree). Data are available under the terms of the Creative Commons Attribution 4.0 International license (CC-BY 4.0). Zenodo. BFPT-TPACK-CLMS-ExtData. https://doi.org/10.5281/zenodo.21245178 ( Lai, 2026b). This project contains the following extended data:
• Questionnaires.docx (TPACK and Big Five Personality Traits Questionnaires) • Supplementary Tables.docx (Containing 3 supplementary tables, (1) Constructs’ Indicators Outer Loadings, (2) PLSpredict MV Summary, (3) Hypotheses and Summary of Findings) Data are available under the terms of the Creative Commons Attribution 4.0 International license (CC-BY 4.0). The scope of this study does not fall under clinical trials, animal research, observational studies or qualitative research. As such, reporting guidelines such as CONSORT, ARRIVE, STROBE, COREQ and SRQR are not applicable.
