# Supplementary material for: Integrating Big Five Personality Traits and TPACK: A Pedagogical Framework for Collaborative Learning Management System (C-LMS) in Higher Education
Source: F1000Res. 2026 Sep 25;15:1288. Originally published 2026 Aug 4. [Version 2] doi: 10.12688/f1000research.183549.2 (PMC13646544; doi:10.12688/f1000research.183549.2)
Supplement: Peer review history

# Reviewer report: Reviewer response for version 2

**DOI:** 10.5256/f1000research.210310.r524471

**Published:** 2026-10-07

**Authors:** A Shobana Devi

**Affiliations:** 1 Department of Data Science and Business Systems, SRM Institutte of Science and Technology, Chennai, Tamil Nadu, India

As the responses to all review comments are fixed and clear explanation is given as responses document. The article has been accepted to index in this journal.

Is the work clearly and accurately presented and does it cite the current literature?

Yes

If applicable, is the statistical analysis and its interpretation appropriate?

Yes

Are all the source data underlying the results available to ensure full reproducibility?

Yes

Is the study design appropriate and is the work technically sound?

Partly

Are the conclusions drawn adequately supported by the results?

Partly

Are sufficient details of methods and analysis provided to allow replication by others?

Partly

Reviewer Expertise:

Machine Learning, Deep Learning, Artificial Intelligence

I confirm that I have read this submission and believe that I have an appropriate level of expertise to confirm that it is of an acceptable scientific standard.

# Reviewer report: Reviewer response for version 1

**DOI:** 10.5256/f1000research.202604.r508623

**Published:** 2026-09-04

**Authors:** A Shobana Devi

**Affiliations:** 1 Department of Data Science and Business Systems, SRM Institutte of Science and Technology, Chennai, Tamil Nadu, India

This manuscript investigates the relationship between students’ Big Five Personality Traits (BFPT), technology-related Technological Pedagogical Content Knowledge (TPACK), and continued use of technology within a proposed Collaborative Learning Management System (C-LMS). The study uses an experimental online-course setting involving 121 students from a local private university. The authors combine a Big Five personality measure with selected technology-related TPACK constructs and analyse the proposed structural model using Partial Least Squares Structural Equation Modelling (PLS-SEM). Academic performance is additionally assessed using pre- and post-module knowledge-checking tests.

 The topic is relevant and timely. The manuscript has a potentially useful contribution because it examines TPACK from the student perspective and combines it with personality traits in a collaborative virtual-learning environment. The article also makes a useful effort to provide underlying questionnaire and academic-performance data, supplementary tables, and the questionnaires used in the study.

 The manuscript is generally well organized, and the statistical analyses are substantial. The reported measurement-model results indicate generally acceptable internal consistency, convergent validity and discriminant validity, while the structural model provides evidence for several of the proposed relationships.

 However, I recommend **major revision before the manuscript can be considered scientifically sound**. The principal concerns relate to the interpretation of causality, the study design and lack of a control/comparison group, sample size and sampling strategy, clarity and consistency of the statistical reporting, the operationalization of academic performance, and several aspects of reproducibility.

 Study design – **Partly**

- Clarify the study design and sampling procedure.
- Justify the sample size of 121 participants.
- Clearly acknowledge the absence of a control group and avoid strong causal claims.

Methods and replication – **Partly**

- Provide more details on the C-LMS intervention, assessment procedure, and questionnaire modifications.
- Clearly describe data collection, scoring, and data-analysis procedures.
- Clarify how participants were assigned to groups.

Conclusions – **Partly**

- Moderate claims about the C-LMS improving academic performance.
- Clearly distinguish between **association and causation**.
- Ensure conclusions are limited to the findings from the single-institution sample.

**Key revisions that must be addressed**

1. Clarify the study design and control/comparison group.
2. Correctly interpret the statistical results.
3. Strengthen methodological details for replication.
4. Moderate causal and generalisation claims.

Is the work clearly and accurately presented and does it cite the current literature?

Yes

If applicable, is the statistical analysis and its interpretation appropriate?

Yes

Are all the source data underlying the results available to ensure full reproducibility?

Yes

Is the study design appropriate and is the work technically sound?

Partly

Are the conclusions drawn adequately supported by the results?

Partly

Are sufficient details of methods and analysis provided to allow replication by others?

Partly

Reviewer Expertise:

Machine Learning, Deep Learning, Artificial Intelligence

I confirm that I have read this submission and believe that I have an appropriate level of expertise to confirm that it is of an acceptable scientific standard, however I have significant reservations, as outlined above.

# Response

**Published:** 2026-09-09

**Authors:** Sook Ling Lew

**Affiliations:** Multimedia University, Malaysia

Study design

- Clarify the study design and sampling procedure.
  - Clarification is added, "3.3 Research Design
    The current research is conducted through a quantitative research design in which numerical data and statistical analysis were utilised to test the formulated hypotheses. Specifically, the current research employed a single-group quasi-experimental pretest-posttest design with the same group of participants (Capili & Anastasi, 2024). Participants are assessed before and after the intervention, and the intervention's effect is determined by the difference between pretest and posttest results."
- Justify the sample size of 121 participants.
  - Justification is added, "According to Kock and Hadaya's Inverse Square Root Method for 80% power at *α=0.05*, the path coefficient (p _min_) in the current study is 0.370, and the required minimum sample size is 58 respondents (Putu Gede Subhaktiyasa, 2024). Therefore, the research involved 121 local private university students enrolled in the online course via the proposed C-LMS, which is sufficient to meet the inverse-square-root method requirement."
- Clearly acknowledge the absence of a control group and avoid strong causal claims.
  - It is addressed by adding, "In the meantime, the current research focuses solely on a single-group quasi-experimental design, in which the control group is absent throughout the experimental phase. Thence, the experiment might refrain from inferring the definitive cause-and-effect relationships from a single-group experimental setting." under 5.2 Limitations and Recommendations.

Methods and replication

- Provide more details on the C-LMS intervention, assessment procedure, and questionnaire modifications.
  - Details are added, " The questionnaire is modified to fit the use case and application in the current research. The questions were originally altered from the teacher's perspective to the student's, providing insights from other points of view regarding the integration of technology in the modern classroom."
- Clearly describe data collection, scoring, and data-analysis procedures.
  - Description is added, Throughout the experiment phase, the participants engaged with the C-LMS across enrolled coursework modules. These modules consist of collaborative tasks, such as knowledge sharing and collective problem-solving, that require participants to interact with their peers in small groups. These participants were assessed based on their academic outcome group-based activities through the C-LMS dedicated assessment modules. The assessment modules included a 5-question quiz evaluating participants' prior knowledge and exposure to the module's content. Upon completing the module, participants were asked to take a 5-question quiz to assess their understanding of the topic covered in the modules they enrolled in. The maximum score for these quizzes was 10, where each question was worth 2 marks. These marks were recorded as input for a paired-samples t-test to identify differences in participants' pre- and post-knowledge-checking test performance. As for the BFI-20 and TPACK questionnaires, a 5-point Likert scale is used, ranging from "5 = Strongly Agree" to "1 = Strongly Disagree". Odd Likert scales are preferred for better reliability, validity, discrimination, precision, and stability than even Likert scales (Mohd Rokeman, 2024). Hence, the 5-point Likert scale is ideal and favours the current research."
- Clarify how participants were assigned to groups.
  - Clarification is added, " The students are randomly divided into small groups of 4. The groups were formed regardless of their gender, race, ethnicity, nationality, and personality traits. The students will be assigned to a group once they enroll in the dedicated online course. The C-LMS automatically divides these students into different groups equally based on the total number of enrolled students."

Conclusions

- Moderate claims about the C-LMS improving academic performance.
  - It is addressed by adding, "The current research indicates a significant relationship between C-LMS and academic performance, with engagement within the C-LMS positively associated with students' academic performance, suggesting that collaborative features are associated with higher academic outcomes."
- Clearly distinguish between **association and causation**.
  - Added, "In the meantime, the current research focuses solely on a single-group quasi-experimental design, in which the control group is absent throughout the experimental phase. Thence, the experiment might refrain from inferring the definitive cause-and-effect relationships from a single-group experimental setting."
- Ensure conclusions are limited to the findings from the single-institution sample.
  - Added, "Besides, the participants in the current research are based on the same higher education institution. Henceforth, the result of the current research might not be sufficient to reflect and represent the university student population as it is based on a single-institution sample."

# Reviewer report: Reviewer response for version 1

**DOI:** 10.5256/f1000research.202604.r513641

**Published:** 2026-09-03

**Authors:** Maizatul Akmar Ismail

**Affiliations:** 1 Universiti Malaya, Kuala Lumpur, Malaysia

This is a valuable study that offers an integration of the Big Five personality traits, TPACK, and collaborative learning within a C-LMS environment. The research questions are relevant, the conceptual framework is well motivated, and the empirical analysis provides insights for technology-enhanced higher education. I particularly appreciate the student-centred perspective and the practical implications for collaborative learning. Overall, the manuscript is structured and makes a meaningful contribution. My main concern relates to reproducibility. Although datasets are made available, clearer replication details, including the full analysis procedure, software settings, instruments, and C-LMS implementation specifications, would strengthen confidence in reproducing the findings.

Is the work clearly and accurately presented and does it cite the current literature?

Yes

If applicable, is the statistical analysis and its interpretation appropriate?

Yes

Are all the source data underlying the results available to ensure full reproducibility?

Partly

Is the study design appropriate and is the work technically sound?

Yes

Are the conclusions drawn adequately supported by the results?

Yes

Are sufficient details of methods and analysis provided to allow replication by others?

Yes

Reviewer Expertise:

Information Systems, Data Science

I confirm that I have read this submission and believe that I have an appropriate level of expertise to confirm that it is of an acceptable scientific standard.

# Response

**Published:** 2026-09-14

**Authors:** Sook Ling Lew

**Affiliations:** Multimedia University, Malaysia

Although datasets are made available, clearer replication details, including the full analysis procedure, software settings, instruments, and C-LMS implementation specifications.

 Justification as follows: 

 "3.3 Research Design

 The current research is conducted through a quantitative research design in which numerical data and statistical analysis were utilised to test the formulated hypotheses. Specifically, the current research employed a single-group quasi-experimental pretest-posttest design with the same group of participants (Capili & Anastasi, 2024). Participants are assessed before and after the intervention, and the intervention's effect is determined by the difference between pretest and posttest results. Moreover, confirmatory factor analysis is conducted through PLS-SEM path modeling via SmartPLS 4.0."

 "According to Kock and Hadaya's Inverse Square Root Method for 80% power at *α=0.05* , the path coefficient (p _min_) in the current study is 0.370, and the required minimum sample size is 58 respondents (Putu Gede Subhaktiyasa, 2024). Therefore, the research involved 121 local private university students enrolled in the online course via the proposed C-LMS, which is sufficient to meet the inverse-square-root method requirement."

 "Throughout the experiment phase, the participants engaged with the C-LMS across enrolled coursework modules. These modules consist of collaborative tasks, such as knowledge sharing and collective problem-solving, that require participants to interact with their peers in small groups. These participants were assessed based on their academic outcome group-based activities through the C-LMS dedicated assessment modules. The assessment modules included a 5-question quiz evaluating participants' prior knowledge and exposure to the module's content. Upon completing the module, participants were asked to take a 5-question quiz to assess their understanding of the topic covered in the modules they enrolled in. The maximum score for these quizzes was 10, where each question was worth 2 marks. These marks were recorded as input for a paired-samples t-test to identify differences in participants' pre- and post-knowledge-checking test performance. As for the BFI-20 and TPACK questionnaires, a 5-point Likert scale is used, ranging from "5 = Strongly Agree" to "1 = Strongly Disagree". Odd Likert scales are preferred for better reliability, validity, discrimination, precision, and stability than even Likert scales (Mohd Rokeman, 2024). Hence, the 5-point Likert scale is ideal and favours the current research."
